# Barriers to diagnosing and treating vulval lichen sclerosus: a survey study

**DOI:** 10.3399/BJGP.2024.0360

**Published:** 2025-02-25

**Authors:** Arabella Crew, Rheanne Leatherland, Louise Clarke, Caroline Owen, Rosalind C Simpson

**Affiliations:** University of Nottingham, Nottingham.; Centre of Evidence Based Dermatology, University of Nottingham, Nottingham.; Centre of Evidence Based Dermatology, University of Nottingham, Nottingham.; East Lancashire Hospitals NHS Trust, Blackburn and Burnley.; Centre of Evidence Based Dermatology, University of Nottingham, Nottingham.

**Keywords:** dermatology, general practice, lichen sclerosus, primary health care, qualitative research, vulval lichen sclerosus

## Abstract

**Background:**

Vulval lichen sclerosus (VLS) is a chronic inflammatory condition that is frequently misdiagnosed and under-recognised. To date, qualitative research has focused on lived experience of VLS, with women attributing diagnostic delay to poor interactions with healthcare professionals (HCPs), often due to lack of knowledge. In the UK, women with VLS are most likely to present to primary care.

**Aim:**

To establish HCPs’ perspectives on the identification, management, and education of vulval skin disease, with a focus on VLS.

**Design and setting:**

A mixed-methods study survey undertaken across the UK.

**Method:**

HCPs were recruited through opportunistic sampling. The survey was distributed via email and WhatsApp through professional networks and in hard-copy format at events, and completed between 1 November 2023 and 14 December 2023. Data were analysed using descriptive statistics, Spearman’s rank correlations, and thematic analysis.

**Results:**

Of 122 responders, most were GPs (*n* = 53) and GP trainees (*n* = 59). In total, 37.7% of responders had never participated in teaching or learning on vulval skin disease. Confidence in the identification of vulval skin disease positively correlated with experience, exposure, and female gender, although this last correlation was weak. The top identified barriers to diagnosis and treatment included lack of knowledge, embarrassment, and absence of VLS diagnostic criteria. Almost all participants (97.5%) felt that VLS diagnostic criteria would be helpful in clinical practice.

**Conclusion:**

This study provides insight into the barriers to diagnosing and treating VLS in primary care. HCPs recognise deficiencies in training and referral pathways, and a lack of tools to support VLS diagnosis. Training should include skills to address stigma and embarrassment. This study highlights the importance of developing interventions, such as reproducible diagnostic criteria, to overcome barriers, thereby expediting diagnosis and treatment.

## Introduction

Vulval lichen sclerosus (VLS) is a chronic inflammatory skin condition^1^^,^^2^ affecting up to one in 300 patients referred to dermatology.^3^ VLS has a bimodal distribution, presenting more frequently in prepubertal girls (approximately one in 900) and postmenopausal women (up to three in 100) than in females of other ages.^4^^–^6 However, the exact prevalence and incidence are unknown, with poor recognition and misdiagnosis leading to an underestimation of cases.^1^^,^^7^^,^^8^

Women with VLS typically present experiencing soreness, pruritus, burning, and dryness.^1^^,^^4^^,^^9^ On examination, there is often a whitening of vulval skin, ecchymoses, and fissuring, most commonly in a figure-of-eight pattern in the anogenital area.^1^ This can progress to irreversible changes to the vulval architecture, such as clitoral phimosis, fusion of the labia, and obstruction of the urethra.^4^^,^^10^^,^^11^ VLS impacts daily activities such as toileting and psychosexual functioning^12^^,^^13^ and, further, carries at least a 20-fold relative risk of vulval cancer when compared with data for women without VLS.^14^

Women with VLS most often present to primary care,^1^^,^^9^ where early diagnosis, appropriate treatment, and patient education can improve symptoms, restore quality of life, minimise scarring, and reduce cancer risk.^15^^,^^16^ However, identification and delineation of vulval skin disease, including VLS, can be challenging. Misdiagnosis of VLS as thrush or menopausal changes (among other conditions) is common, leading to a delay in correct diagnosis, effective treatment, and symptom relief.^6^^,^^8^^,^^15^^,^^17^ To the authors’ knowledge, there is no decision aid or criteria to aid diagnosis of VLS in primary care.^18^

In a recent qualitative study,^17^ there was an overarching theme of missed opportunities, with patients reporting barriers to diagnosis, such as dismissal of concerns, lack of HCP knowledge, and receiving an incorrect diagnosis. In addition, two qualitative studies^8^^,^^17^ found that women experience embarrassment and shame due to the nature of VLS and its symptoms, leading to delayed presentation. The study presented here aims to explore possible reasons for these missed opportunities for diagnosis and treatment by investigating the perspectives and experiences of HCPs working in primary care.

**Table:** How this fits in

Previous research has identified a significant diagnostic delay and misdiagnosis of vulval lichen sclerosus (VLS), a condition with which patients most commonly present in primary care. Healthcare professionals (HCPs) in primary care share the concerns of women with VLS, citing frequent misdiagnosis, embarrassment, and lack of knowledge as barriers to diagnosis. In this survey, 37.7% of HCPs reported never having participated in learning on vulval skin disease, be it self-directed or otherwise, and 92.6% of HCPs felt further education would be useful. Key enablers that were identified to facilitate timely VLS diagnosis and treatment included a comprehensive education programme for HCPs, implementation of standardised pathways of care, and development of VLS diagnostic criteria that could be implemented in the primary care workflow.

## Method

A mixed-methods study survey (Supplementary Box S1) consisting of Likert scales, ranking, and free-text questions was distributed to professional networks via email and WhatsApp. Sampling was opportunistic using primarily professional networks of the authors, and local networks, such as Health Education England’s GP training network, Next Generation GP East Midlands, the then-named East Midlands Clinical Research Network, and GP trainees East Midlands WhatsApp group. In addition, paper copies for completion were distributed at regional GP trainee teaching sessions. There was no financial or material incentive for taking part in the survey.

Surveys were completed online (via Jisc — www.jisc.ac.uk) or hard copy between 1 November 2023 and 14 December 2023, and responses were anonymously exported into Excel. Paper copies of the survey were stored in a locked filing cabinet in a secure building, until data was inputted into Excel. These have since been disposed of confidentially. The survey data and analysis are stored on the University of Nottingham’s Microsoft OneDrive, accessible only by user identifiers, and are password protected.

Likert scales between 1 (low) and 5 (high) were used to assess:

- frequency and confidence in examining the vulva;
- confidence identifying, diagnosing, and treating vulval skin disease/VLS;
- amount of teaching/learning, if received; and
- how useful further education and a diagnostic tool would be.

Participants were also asked:

- which diagnostic tool format would be most useful;
- to rank their top three barriers to VLS diagnosis and treatment out of 12 predefined options and a free text box labelled ‘other’; and
- to explain their choices.

In addition to the free-text question to discuss barriers, there was a free-text box to specify any other barriers and another for comments on any topics covered by the survey.

### Data analysis

#### Quantitative data

Demographic and survey data were analysed using descriptive statistics. Spearman’s rank tests calculated using SPSS (version 28), explored potential relationships between participants’ confidence levels and other variables, with a Bonferroni correction adjustment alpha (adjusted *P*-value) of <0.003. A Spearman’s rank of -1 or 1 was considered to constitute a strong relationship, whereas correlations closer to 0 were considered weaker. To analyse the ranking of barriers, a weighted score was calculated. Cases with missing data were excluded from analysis for each variable investigated.

#### Qualitative data

Survey responses were coded in NVivo (version 14.23); descriptive themes were developed inductively^19^ using a semantic approach to thematic analysis, with codes strictly driven by the data. Analysis phases included familiarising with the data, generating codes, and reviewing and defining descriptive themes. Two researchers independently coded the data, with a third researcher comparing the codes and highlighting inconsistencies. Both coders were from medical backgrounds and recognised the influence this might have on data analysis, for example by the unconscious prioritisation of codes within the data that the coders also find clinically relevant. A third researcher, who was from a non-medical background was included to ensure content was accurately recognised. The final coding and descriptive themes were collectively agreed by the three researchers.

## Results

A total of 122 participants completed the survey. Missing data was minimal, comprising twelve responses over three questions. Their characteristics are outlined in Table 1. The majority were female (74.6%), GP trainees (48.4%), or GPs (43.4%); the mean age of all participants was 39.5 years.

**Table 1.** Participant characteristics, *n* = 122

**Characteristic**	
**Gender, *n* (%)**	
Female	91 (74.6)
Male	29 (23.8)
Prefer not to say	2 (1.6)

**Mean age, years**	
All participants	39.5
Females	41.0
Males	35.7

**Primary healthcare profession, *n* (%)**	
GP trainee	59 (48.4)
GP	53 (43.4)
Advanced nurse practitioner	5 (4.1)
Practice nurse	4 (3.3)
Other	1 (0.8)

**Years in the profession, *n* (%)**	
<1	19 (15.6)
1–5	61 (50.0)
6–10	12 (9.8)
11–20	12 (9.8)
>20	18 (14.8)
Mean	5.9

**Ethnicity, *n* (%)**	
**Asian or Asian British**	
Indian	13 (10.7)
Pakistani	12 (9.8)
Bangladeshi	1 (0.8)
Chinese	1 (0.8)
Malaysian	1 (0.8)
Any other Asian background	1 (0.8)
**Black African, Caribbean or Black British**	
African	14 (11.5)
Caribbean	1 (0.8)
Black British	1 (0.8)
Any other mixed or multiple ethnic background	1 (0.8)
**Mixed or multiple ethnic groups**	
Any other mixed/multiple ethnic background	1 (0.8)
White and Black African	1 (0.8)
UK/South American	1 (0.8)
**White**	
English/Welsh/Scottish/Northern Irish/British	66 (54.1)
Any other White background	4 (4.1)
Prefer not to say	3 (2.5)

**Figure 1.**
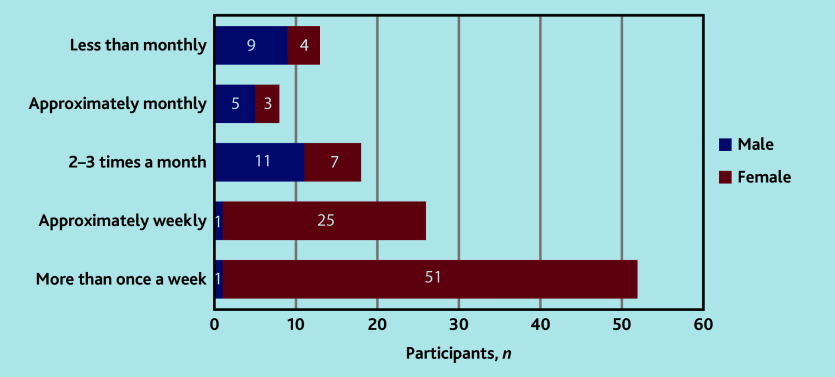
Participant frequency of performing an examination of the vulva.

Female participants saw female genitalia and performed vulval clinical examination more frequently than males: most female HCPs reported performing examinations of the vulva ‘more than once a week’, whereas most male HCPs reported doing this ‘2–3 times a month’ (Figure 1).

### Education and training on vulval skin disease

In total, 44 (36.1%) participants responded ‘yes’ to receiving organised teaching and 23 (18.9%) responded ‘yes’ to participating in self-directed learning relating to vulval skin disease, with a mean teaching duration of 2 hours. However, 37.7% of participants reported having neither received organised teaching nor participated in self-directed learning. In addition, there were a high number (20.5%) of participants who responded ‘not sure’ to whether they had received organised teaching or self-direct learning. Most participants felt that further education on VLS would be helpful, with 92.6% of participants reporting it as ’fairly useful’ or ’very useful’ overall (data not shown).

### Confidence in identifying and managing vulval skin conditions

Table 2 displays the confidence of participants in aspects of diagnosis and management of vulval skin disease, as measured on a Likert scale (1 = ‘not confident at all’, 5 = ‘very confident’). An increasing mean value correlates with higher levels of confidence in this skill. The confidence intervals for the mean Likert responses of males and females do not overlap when participants rated their confidence in the following skills:

- examining the vulva;
- identifying vulval skin disease on examination; and
- identifying VLS on examination.

This suggests a statistically significant difference (p < 0.05) between male and female participants in their self-reported confidence levels for these skills, as measured by the Likert scale. Initiating treatment for patients with VLS was the only skill for which there was no significant difference in confidence between males and females (Table 2).

**Table 2.** Participant skill confidence levels

**Skill**	**Mean Likert scale response^a^ (95% CI)**	
	**Females**	**Males**
Examining the vulva	3.96 (3.79 to 4.12)	3.14 (2.73 to 3.54)
Identifying vulval skin disease on examination	3.91 (3.43 to 3.80)	2.79 (2.45 to 3.14)
Identifying VLS on examination	3.60 (3.43 to 3.78)	2.97 (2.64 to 3.29)
Initiating treatment for VLS	3.36 (3.09 to 3.64)	2.83 (2.45 to 3.21)

a *1= not confident at all; 5 = very confident. VLS = vulval lichen sclerosus.*

### Helpfulness of diagnostic criteria and preferred format

In total, 97.5% of responders felt that clear VLS diagnostic criteria would be helpful, comprising of 68.8% of responders that selected ‘very helpful’ and 28.7% ‘fairly helpful’. The other 2.5% of responders felt that diagnostic criteria would be ‘neither helpful nor unhelpful’ (data not shown). An integrated template (55.0%) in a clinical system (for example, Ardens F12) was the most preferred tool format, followed by weblink (32.2%).

### Correlations between HCP confidence and characteristics

Spearman’s rank correlations were used to explore the relationships between HCP confidence in their skills and their personal or job characteristics; these are shown in Table 3. Frequency of seeing female genitalia as part of the HCP’s role and confidence in identifying vulval skin disease had the strongest positive correlation (0.616) (Table 3). Correlations between female gender and confidence identifying VLS (0.254), examining the vulva (0.313), and identifying vulval skin disease (0.315) were most weakly correlated (Table 3). All correlations in Table 3 were statistically significant, with an adjusted *P-*value (Bonferroni correction) of <0.003.

**Table 3.** Correlations between HCP confidence and personal or job characteristics

**Correlation factors**	**R**
Frequency of seeing female genitalia as part of HCP’s routine clinical practice and confidence in identifying vulval skin disease	0.616
Frequency of examining the vulva and confidence in examining the vulva	0.505
Participant’s female gender and confidence in examining the vulva	0.313
Participant’s female gender and confidence in identifying vulval skin disease	0.315
How long the participant had been in the job role and confidence in examining the vulva	0.408
How long the participant had been in the job role and confidence in identifying vulval skin disease	0.483
Frequency of seeing female genitalia as part of HCP’s routine clinical practice and confidence in identifying VLS on examination	0.458
Participant’s female gender and identifying VLS on examination	0.254

*HCP = healthcare professional; VLS = vulval lichen sclerosus.*

### Participants’ opinions on the barriers to diagnosis and treatment of VLS

**Figure 2.**
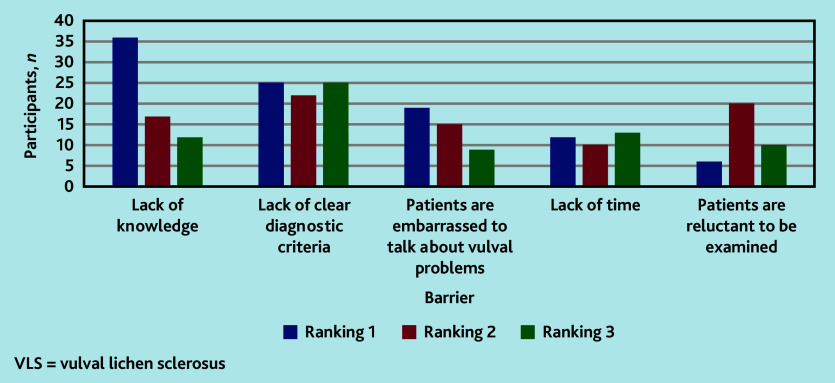
Participants’ opinions on the barriers to diagnosis and treatment of VLS.

Participants were asked to rank the top three barriers to the diagnosis of VLS from a list of 12 predefined options; they were also given a free-text option (Supplementary Box S1 and S2). Participants’ highest-ranking barriers to diagnosis and treatment of VLS were lack of knowledge, lack of clear diagnostic criteria, and patients being embarrassed to talk about vulval problems (Figure 2). Lack of knowledge was the most consistently selected and highest-ranked barrier (Figure 2).

### Qualitative data analysis

**Figure 3.**
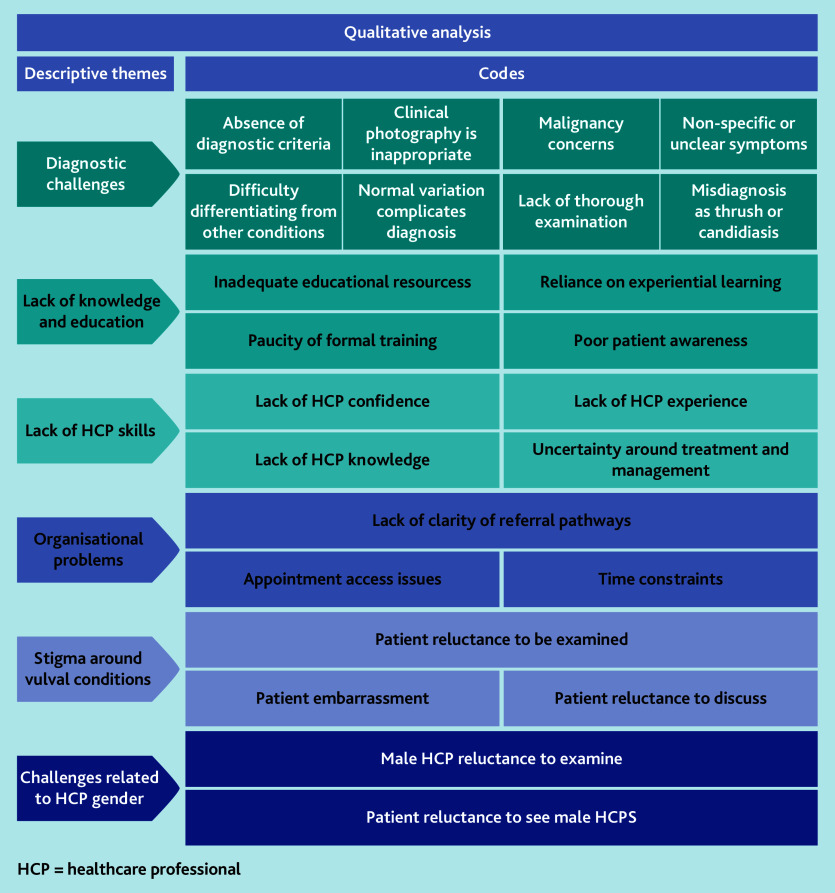
Descriptive themes and codes.

Analysis of the free-text data resulted in six descriptive themes being developed from 24 inductive codes (Figure 3).

#### Diagnostic challenges

Participants expressed confusion over whether diagnostic criteria were available and whether referral was needed to confirm VLS diagnosis:

*‘I diagnose based on symptoms and examination findings. Not aware of diagnostic criteria’*

(female GP, age 40–49 years, 1–5 years in role).

Such uncertainty may have contributed to delays in diagnosis and misdiagnosis that were observed by clinicians:

*‘Vulval symptoms are often treated as* Candida *for years’*

(female GP, age 50–59 years, >20 years in role).

This was exacerbated by reports of VLS presentation being unclear, due to the variation in normal vulval appearance:

*‘Sometimes it is obvious, but other times I’m not sure if it is postmenopausal changes’*

(female GP trainee, age 30–39 years, 1–5 years in role).

HCPs recognised there was uncertainty among the patients themselves, particularly when recognising VLS symptoms and signs versus normal menopausal changes:

*‘Women present very late with scarring. They think it’s normal as they age’*

(female GP, age 50–59 years, >20 years in role).

#### Lack of knowledge and education

According to participants, there was a lack of formal training and limited experience around vulval skin conditions among HCPs, resulting in reduced confidence in treating and diagnosing them:

*‘Many GPs do not do additional obstetrics and gynaecology training now so are not confident in diagnosing vulval conditions or gynae problems’*

(female GP, age 50–59 years, >20 years in role).

Participants felt that they relied on exposure to the condition in practice for learning:

*‘I have not had formal teaching on this but did spend some time in vulval dermatology clinics during GP training; without this, I would have significantly less confidence’*

(male GP, age 18–29 years, 1–5 years in role).

#### Lack of HCP skills

Participants described confusion about whether the treatment and management of vulval conditions fell under primary or secondary care, and also highlighted their caution when using topical steroids:

*‘I’m not sure if all patients need to see a consultant prior in order to have diagnosis confirmed or if* [it is] *OK to trial treatment’*

(female advanced nurse practitioner [ANP], age 50–59 years, 1–5 years in role).

*‘There is always a bit of a reluctance to commence topical steroids and knowing the potency* [that is] *appropriate’*

(female GP trainee, age 30–39 years, 1–5 years in role).

Participants also reported a preference to refer to other professionals, instead of diagnosing themselves, with some citing concerns over missing malignancy:

*‘*[…] *I am more likely to refer to specialists for confirmation if I am unsure about the criteria, or how to safely treat’*

(female GP, age 30–39 years, 1–5 years in role).

#### Organisational problems

A lack of resources in general practice was felt to be a barrier to diagnosis by participants. This included there being no easy access to educational resources, particularly those with clinical photographs, hindering accurate identification of vulval skin disease. A lack of female GPs to facilitate appointments was reported; this was a barrier as it was reported that patients often preferred to see a female doctor for vulval issues, resulting in an increased burden of work on female clinicians:

*‘In my practice, an overall lack of female GPs creates a barrier as, often, these patients wish to see a female doctor and so there is a burden of work on the available female doctors’*

(female GP, age 50–59 years, >20 years in role).

Referral to specialists may also be delayed by unclear referral pathways:

*‘I think some clear indication of the pathways locally would be helpful to ensure timely referral to specialists’*

(female GP trainee, age 30–39 years, 1–5 years in role).

Furthermore, a lack of adequate time in appointments was felt to contribute to incorrect and missed diagnoses:

*‘GP is very busy, with a large burden of work, which can at times mean a quick ‘maybe thrush, treat as thrush first’ rather than examination’*

(female GP, age 50–59 years, >20 years in role).

#### Stigma around vulval conditions

Participants recognised the challenges that patients faced in seeking help for vulval conditions, adding further complexities to diagnosis. These challenges included a reluctance to discuss sensitive information or be examined, with embarrassment felt to be an underlying reason for this:

*‘Patients do find it difficult to talk about private issues relating to their genitals and I feel extra consultation skills are required* […]*’*

(female ANP, age 30–39 years, 1–5 years in role).

*‘*[…] *They may feel embarrassed to come to see doctors about this’*

(male GP trainee, age 18–29 years, 1–5 years in role).

#### Challenges related to HCP gender

A lack of experience in vulval conditions for male clinicians was reported, caused by both a patient reluctance to be examined by male clinicians and a reluctance from male clinicians themselves to undertake female genital examination. Male participants described patient preference for female clinicians when genital examination was a possibility:

*‘Female patients are reluctant to be examined by male doctors for intimate examinations’*

(male GP trainee, age 18–29 years, 1–5 years in role).

Female participants, similarly, reported a lack of examination done by their male colleagues and a tendency for them to refer elsewhere, which could leave them lacking experience and confidence in this area:

*‘I find that male colleagues often refer patients with these issues to see female clinicians, which may delay the diagnosis/mean they see less* [sic] *so aren’t as confident in the diagnosis’*

(female GP trainee, age 18–29 years, 1–5 years in role).

## Discussion

### Summary

This study explores the barriers to diagnosing and treating VLS from the perspective of HCPs in primary care. Several barriers were described in the data, including lack of confidence and experience in the skills essential for VLS diagnosis. Stigma around vulval conditions also emerged as a theme in the qualitative data. Participants recognised that patients could be reluctant to seek help due to embarrassment; a lack of awareness among patients themselves of what is normal was also cited as a barrier to diagnosis.

In terms of HCPS, infrequent exposure to vulval examinations and time spent in the job role were correlated with a lack of confidence. Female participants performed vulval examinations more frequently than males, but correlation between confidence in the skills associated with VLS diagnosis and female gender was weak. The relative inexperience of male clinicians translated into fewer opportunities for experiential learning, resulting in poorer confidence in diagnostic skills. This led to a cycle: clinicians with poor confidence might be more reluctant to examine, resulting in no experiential learning and, as such, inexperience. There was a lack of relevant education and training among the study participants, which is likely to contribute to poor confidence. Lack of knowledge and educational opportunities were consistently recognised throughout the qualitative data. More than a third of participants had neither received teaching nor participated in relevant self-directed learning in vulval skin disease; however, results suggested that, regardless of the teaching that had been received, participants felt that more education could be useful.

Female participants rated themselves as statistically significantly more confident than males at examining the vulva, identifying vulval skin disease, and initiating treatment for VLS. Although statistically significant, the correlation between female gender and confidence in these skills was, however, weak. Participants acknowledged the fact that opportunities for male clinicians to examine were limited, due to patients feeling more at ease and less embarrassed when examined by female clinicians. Participants recalled patients who did not want to be examined by male clinicians, and male clinicians who were reluctant themselves — and, thereby, felt deskilled due to limited exposure. As a result, participants reported cross-referral to female colleagues in primary care, potentially impacting patient care by delaying diagnosis and discouraging patients from seeking examination with male clinicians further.

Participants consistently highlighted challenges diagnosing VLS, expressing uncertainty over the differential diagnoses, management guidelines, and whether diagnostic criteria were available. Variation in VLS presentation, normal variation, and absence of diagnostic criteria were felt to make diagnosis difficult. In addition, organisational challenges within primary care and the interface with secondary care — for example, time constraints and referral pathways — were also found to pose barriers to VLS diagnosis and treatment. Supporting HCPs in the diagnosis of VLS via a diagnostic tool was considered a helpful suggestion. Almost all participants felt that the provision of clear diagnostic criteria for VLS would be helpful, particularly if a template was integrated into the existing clinical system. Three participants said the criteria would be ‘neither helpful nor unhelpful’, but all three examined female genitalia as part of routine practice either weekly or more often; this regular exposure may have influenced their view on the helpfulness of diagnostic criteria, as this was positively correlated with confidence levels in identifying VLS.

### Strengths and limitations

This study is, to the authors’ knowledge, the first to explore the perceptions of HCPs on VLS treatment and diagnosis. A strength of this study is its singularity in collecting quantitative data on the confidence of clinicians in diagnosing and treating VLS, as well as their perspectives on doing so. It complements the existing literature on the shortcomings in diagnosing and treating VLS, and provides some explanation for these challenges. Greater depth of insight could have been obtained by conducting a study with direct contact with participants — that is, an interview or focus group study. Using a survey meant that limited qualitative data could be gathered via free-text responses; however, it was a practical method given the authors’ resources and it provided novel viewpoints.

Participants mainly comprised GPs and GP trainees in the Midlands, UK. This limits the generalisability of the findings and did not allow for analysis between different clinicians or different regions. This is reflective of the opportunistic recruitment, which was largely reliant on the professional networks of the authors. To increase the sample size, the advertisement was sent to six different WhatsApp groups, email lists, and the local TeamNet site; it is, therefore, difficult to estimate the number of eligible HCPs that viewed the advertisement and, subsequently, the response rate. The authors recognise that inaccuracies in ethnicity recording were likely to have been introduced within the study due to a lack of clarity in the survey categories for ethnicity. The data we collected suggested the sample is broadly representative of the UK GP workforce with regards to ethnicity and age, although there were more than three times the number of female participants than males. There are more female than male GPs in the UK as of 2024,^22^ but it should be noted that the national ratio is smaller.

GPs and GP trainees constituted most of the responders, which was not only due to the authors’ professional networks, but also likely due to their more active role in diagnosing skin disease. However, to the authors’ knowledge, this is the first study to quantify the confidence in clinical skills relevant to vulval skin disease and women’s health, correlating them with participant characteristics.

Twelve responses over three questions were missing due to participants not answering every question. Despite this, saturation of themes was reached, and expectation of sample size was exceeded.

### Comparison with existing literature

HCPs echoed the experiences of women with VLS in the qualitative literature. Themes and subthemes around diagnosis identified in this study overlap with those highlighted in the existing qualitative literature that explores the experience of patients: patients and HCPs found a lack of HCP knowledge, embarrassment, frequent misdiagnosis as thrush, and potential malignancy concerning. There is an unmet need to educate HCPs in vulval skin disease and the lack of knowledge leads to misdiagnosis. Women’s health and dermatology are known, historically, to have low priority in medical school curricula.^20^^,^^21^ Both patients and HCPs have acknowledged the frequent misdiagnosis of VLS as vulval candidiasis and the effect of embarrassment on consultations. Participants in the study presented here felt that additional education on vulval skin disease in primary care would be beneficial, substantiating the calls for HCP education to be a priority, as highlighted in previous studies.^8^^,^^17^^,^^23^ The gender confidence gap has been well recognised in the literature so the correlation between female gender and confidence in relevant skills may be underestimated.^24^

Variation in practice in facilitating testing for sexually transmitted infections^25^ and female pelvic examinations^26^ has been described in primary care, with similar barriers to those identified by the study presented here. In a qualitative study of the use of female pelvic examinations by GPs,^26^ the authors found similar findings in capability (education and confidence), opportunity (time constraints), and motivation (concerns about embarrassing patients).

This survey identified that almost all participants would find diagnostic criteria helpful, which mirrors the findings of a James Lind Alliance Priority Setting Partnership.^27^ This partnership established diagnostic criteria as the second-most important research priority for lichen sclerosus.

### Implications for research and practice

Embarrassment is more common in women and in consultations requiring intimate examinations, leading to medical avoidance.^28^ Shame and embarrassment are associated with negative health outcomes and mental health conditions;.^29^^,^^30^ these feelings are a reason for delayed presentation of vulval conditions, as well as a hindrance to communication.^17^ Addressing embarrassment, shame, and stigma in relation to vulval symptoms is something that requires change at a societal level; however, HCPs also have an important role to play and can aim to address it in their individual interactions with patients. Acknowledgement of stigma and recognition of embarrassment may help validate patients’ feelings and improve their experience.^30^ Integrating education on consultation skills into future vulval skin disease teaching initiatives could further enhance the patient–doctor relationship. The COM-B behaviour change model, which was used in the two studies^25^^,^^26^ on STI testing and female pelvic examinations that were previously mentioned, identifies the component(s) — that is, behaviour, capability, opportunity, or motivation — that must be modified for an intervention to be successful;^31^ this could be considered in future implementation of changes for vulval examination.

The lack of patient awareness of what is normal further emphasised the importance of educational initiatives — not only for HCPs, but also for the public. Participants felt that educating patients and raising awareness of symptoms of VLS may increase the likelihood of seeking help, thereby expediting diagnosis.

Gaps identified in education on vulval skin disease provide an opportunity for attainable improvement. Developing a comprehensive education programme for integration into the training of HCPs in primary care that incorporates consultation skills, presentation of vulval disease, and differential diagnoses could improve patient outcomes. The fact that participants thought that more teaching could be useful reinforces the need for targeted, easily accessible, relevant educational resources for primary care clinicians. Differences in the appearance of VLS in people of colour was also mentioned in the survey data mentioned in the discussion:

*‘Sometimes it is obvious, but other times I’m not sure if it is postmenopausal changes. It is also a lot harder in black/brown skin.’*

(female GP trainee, age 30–39 years, 1–5 years in role).

Future educational resources should include diverse photography and information to ensure equality.

Research into clear, validated, and evidenced-based diagnostic criteria that are suitable for use in primary care should be prioritised. Future research in this area should also focus on the experiences of other types of HCP in primary care.

Although it is difficult to create time in primary care, referral pathways should be well established and standardised, including advice and guidance when appropriate. Implementation of standardised pathways of care, including guidance on which speciality to refer to, as well as accessing advice and guidance services more frequently, would streamline the patient journey. Criteria for advice and guidance or referral should be clear and bespoke for patients presenting with vulval issues, especially given the challenges of teledermatology in this group. Aspirational standards of care have been published by the British Society for the Study of Vulval Disease and should be considered when such pathways are being developed.^32^ Finally, future research could focus on how VLS diagnostic criteria could be integrated into the primary care workflow.

## Data

The survey data is available from the University of Nottingham data repository: http://doi.org/10.17639/nott.7519
